# The Hospital. Nursing Section

**Published:** 1905-07-08

**Authors:** 


### The Hospital.

### Nursing Section.

Contributions for this Section of "The Hospital" should be addressed to the Editor, "The Hospital" Nursing Section, 28 & 29 Southampton Street, Strand, London, W.C.

No. 980.—Vol. XXXVIII. SATURDAY, JULY 8, 1905.

### Notes on News from the Nursing World.

### ROYAL NATIONAL PENSION FUND FOR NURSES.

Sir John Ure Primrose has been elected a Member of the Council of the Royal National Pension Fund for Nurses, and has accepted office. We are confident that the nurses of Scotland will welcome the election of a member for Scotland on the Pension Fund Council. The election of the Lord Provost of Glasgow as the representative of nursing in Scotland cannot fail to draw the Scottish nurses more closely to the Fund, for it must appeal more directly to the Scotch feeling of clannishness, than which no lever is probably more powerful and enduring.

### LADY PIGGOTT.

There is one distinction in the list of Birthday Honours which is of special interest to the nursing world. The King has conferred the dignity of Knight Bachelor upon Mr. Francis Taylor Piggott, Chief Justice of the Supreme Court of the Colony of Hongkong. Sir Francis Piggott is the husband of the indefatigable lady who originated and founded the Colonial Nursing Association, and the recognition of his services to the State will cause many congratulations to be offered to her. It was while her husband was fulfilling the duties of Procureur and Advocate-General in Mauritius that Mrs. Piggott passed through the personal experience which so painfully impressed her with the need of trained nurses in the Crown colonies, with the result that when she returned to England she set herself to the task of supplying the want. How admirably she succeeded, in the face of much discouragement and many difficulties, is well known to those of our readers who are familiar with the history of the Association which was published a few years ago in our columns. Lady Piggott, although unable in present circumstances to take an active part in the work of the Association, remains its honorary Vice-President.

### THE NEW SUPERINTENDENT OF THE JUBILEE INSTITUTE.

The only regret to which the appointment of Miss Amy Hughes as general superintendent of Queen Victoria's Jubilee Institute for Nurses, announced by us last week, will give rise is that it necessarily brings to a close the excellent work she has been doing for the past four years as superintendent of County Nursing Associations. Twelve months ago we published a full account of the progress of this movement, which Miss Hughes has developed with unsparing energy and remarkable tact. One of the numerous members of the Nightingale School who have distinguished themselves, her experience of all branches of nursing, and her success in the various positions she has filled are a guarantee that her new duties as head of the Jubilee Institute will be discharged in a satisfactory manner. Under her auspices there can be no doubt that it will increase in usefulness and that the widest sympathy will be shown to all who are associated with it and bear the honoured title of Queen's Nurses. Miss Hughes is to read a paper at the Church Congress at Weymouth in October on "Town and Village Nursing."

### MISS PETER'S TESTIMONIAL FUND.

We are asked to announce the fact that a Fund has been started to present Miss Peter, who has recently resigned the post of General Superintendent of Queen Victoria's Jubilee Institute for Nurses, with a suitable testimonial from the Queen's Nurses, past and present. Notices are being sent, as far as possible, to all whose names are or have been on the "Roll of Queen's Nurses." The addresses of many ex-Queen's Nurses are not, however, known. Miss Mantell, who is acting as Honorary Treasurer, will be glad to receive subscriptions at 54 Knatchbull Road, Camberwell, London, S.E., before September 1st.

### THE NURSES OF MILLBANK HOSPITAL.

The staff of nurses under Miss Beatrice Jones at the new Military Hospital at Millbank, which was visited by the King and Queen on Saturday afternoon, is at present very small, but it will be increased from time to time as occasion requires. A considerable period, however, must elapse before it reaches its full complement of forty nurses, for the home which will stand immediately behind the main block of the building, has yet to be built, and at present those in attendance on the patients—who were augmented on Monday by a consignment from Malta—have to be accommodated in rooms which will later on be occupied by sick soldiers. The site of the home was inspected by their Majesties.

### NURSES AND MOTOR CARS.

The popularity of motor racing has resulted in the establishment of an encampment just off the cup-race road between Laschamps and Clermont Ferrand of a novel character. Here Baron Henri de Rothschild has provided a staff of nurses, medical appliances, and a large ambulance car in case of emergency. It is a remarkable fact that last week one of the Baron's chaffeurs met with his death in a motor car, through reckless driving, on his return from a visit to the encampment, but too far away from it for the nurses to be able to render assistance.

### FRICTION AT A COTTAGE HOSPITAL.

It seems a pity that a hospital which has done good work for seven years, and derived considerable support from local subscribers, should now be likely to lose some of the latter owing to trouble between the Committee and the nursing staff. This is the condition of affairs at the Cottage Hospital, Carshalton. In January last a complaint was made of the treatment of a patient in the institution. A sub-committee of three was appointed to inquire into the matter, and in the course of their investigations they found that there was so much friction amongst the staff that the interests of the patients could not be properly attended to, and recommended that the resignations of the whole of the staff, matron and nurses, should be requested. This was done, and the matron and the probationer left at the end of their first twelve months at Carshalton. A nurse, however, who had been at the hospital for six years, was asked to remain for a month with the new matron. It is stated that the latter, when spoken to on the subject the night before the nurse's month expired, expressed a desire to retain the nurse as staff nurse, adding that she liked her and had been long enough with her to form an opinion of her capabilities. Notwithstanding this statement the nurse was asked to leave by the next day, and the matron found herself in consequence alone with a new probationer for five operation cases. She has now tendered her resignation, and matters seem to be still in a most unsettled condition, as the only resolution come to at the last stormy meeting was that "it should terminate without any resolution being voted upon." Miss L. Bown, the matron at the outset of the friction, has since been elected, out of 120 applicants, for the post of lady superintendent and matron in charge of a nursing home working under the Holt-Ockley rules.

### THE UNIFORM QUESTION AT SOUTH SHIELDS.

The South Shields Guardians have been discussing the question of uniform. The Hospital Committee recommended in their report that it should be left to the discretion of the probationers whether they should wear their uniform or not when off duty: and in explanation of the recommendation it was stated that the probationers desired to be at liberty to wear ordinary dress when they were cycling. Some of the Guardians demurred to the proposal on the ground that, as two uniforms are provided at the expense of the ratepayers, one for indoor duty and the other for outdoor wear, they ought to be used. We are glad to say that this narrow view of the matter was not taken by a majority, 17 Guardians voting for the adoption of the recommendation of the Committee, and only eight against. There is a good deal to be urged in favour of wearing outdoor uniform, but we certainly think that the less it is made obligatory the better.

### THE TROUBLE AT SCULCOATES INFIRMARY.

With regard to the alleged friction at Sculcoates Workhouse Infirmary, to which we referred in our issue of June 17th, the House Committee, appointed at the suggestion of Dr. Lilley, reported in favour of the dismissal of the nurse who ran away and had in fact discharged herself, explaining her action on the ground that the probationers in the infirmary have "a miserable time." A long and somewhat acrimonious discussion took place at the last meeting of the Sculcoates Guardians, in the course of which somewhat conflicting assertions were made respecting the allegations of the nurse in question, who was herself examined by the committee. Eventually yet another investigation by a special committee was decided upon by the casting vote of the chairman, to be held in the presence of Mr. Bagenal, the Local Government Board inspector. It is to be hoped that the charges which have been freely bandied about, such as favouritism on the part of the superintendent nurse to certain members of the staff, and the refusal of a nurse to write a letter to a friend for a dying inmate of the infirmary, will now be satisfactorily dealt with.

### THE ABSENCE OF THE SECTARIAN SPIRIT IN DISTRICT NURSING.

The Thorpe Hamlet Nursing Association has been cited as an instance of the manner in which organisations of this character are carried on without the slightest difficulty on the broadest unsectarian basis, with a single eye to the service of the poor. This is undoubtedly the case, but the nurse for the Thorpe Hamlet district is a member of the staff of the District Nursing Home in Palace Plain, Norwich, and Miss Sykes, the matron, and the three other nurses attached to the staff, work in other parts of the city under similar conditions. During last year they attended 230 cases in 34 different parishes, making no distinction as to creed. We commend the example of Norwich to the combatants at Chester.

### QUEEN ALEXANDRA'S MILITARY NURSING SERVICE.

The following staff nurses have been confirmed in their appointment to Queen Alexandra's Imperial Military Nursing Service:—Miss L. Belcher, Miss C. T. Bilton, Miss E. C. Fox, Miss M. O'C. McCreery, Miss M. E. Neville and Miss A. Willes.

### DISTRICT NURSING IN WEST LONDON.

Following the annual meeting, in the grounds of Kensington Vicarage, of the Kensington District Nursing Association—whose nurses last year paid no fewer than 27,533 visits—the members of the Hammersmith and Fulham District Nursing Association had their function on the afternoon of Thursday last week. It assumed the form of a garden fête in the grounds of Carnforth Lodge, Hammersmith, at which the Princess Louise and the Duke of Argyll were present, as at Kensington. On this occasion, however, the Princess received purses in aid of the funds of the Association. In reply to a vote of thanks passed to her Royal Highness, the Duke of Argyll expressed the gratification it gave the Princess and himself to be present at such functions, and thereby to further the interests of such admirable institutions.

### NURSES IN THE ORCHESTRA.

On Friday evening the Musical Society of St. Bartholomew's Hospital gave their annual soirée, and there were about 1,800 guests. The orchestra were well led by Mr. Carwardine. A pleasing feature of the arrangements was the admission of nurses to the orchestra, two of them playing first violins. Miss Musson's performance on the harp was much appreciated. Supper was served in the library, and the students were active and agreeable hosts. The quadrangle was prettily lighted with fairylamps. Amongst the items of the programme was the always welcome march from "Tannhäuser," and an amusing song "The Mermaids" received an encore. The National Anthem brought an enjoyable evening to a close.

### WEST HAM AND EAST LONDON HOSPITAL.

About 400 guests attended the "At Home" of the West Ham and East London Hospital on Saturday. The hospital, decorated with flags, palms, and flowers, and newly spring-cleaned and painted, looked its best. Many forms of entertainment were provided for both guests and patients in the men's surgical wards, and the matron and nurses had a very busy time. There was much talk of the preparation for the great effort which is to be made in October on behalf of the extension fund of the institution. We refer to the bazaar at Stratford Town Hall. The matron and nursing staff will have charge of the hospital stall, and we are glad to hear that many leading firms who advertise in our columns are helping Miss Ough in her labour of love.

### MEMORIAL TO THE LATE MISS LUARD.

This week the new portion of the Claughton Convalescent Home at Walton, in connection with the St. Alban's Diocesan Institution for Trained Nurses, was opened as a memorial to Miss M. A. Luard, by whom the institution was founded. As Canon Ingles, vicar of Witham, said at a recent meeting of the subscribers, Miss Luard gave her whole life to the cause of nursing, and then to helping people after the danger of their illness was over. Admiral Sir William Luard, who spoke at the meeting with natural emotion of his sister's work, expressed his gratification that the new portion of the Home was to be called the Luard Hostel, and paid a tribute to the manner in which Miss Leader, the sister in charge of the Claughton Home, discharges her duties. We anticipate with confidence that the opening of the Luard Hostel, in the 32nd year of the existence of St. Alban's Nursing Institution, will prove an addition of great value to the sick poor.

### CLAPHAM SCHOOL OF MIDWIFERY.

The first public examination of the Clapham School of Midwifery took place on Saturday. Though this institution has been training students for 20 years past it has not hitherto been considered desirable to hold examinations as the students were all prepared for the various medical examinations or those of the London Obstetrical Society. But as the latter have now been discontinued and the examinations of the Central Midwives Board are intended specially for midwives practising in England, the authorities of the Clapham School decided to hold a quarterly examination for its own students and others who might care to apply after not less than three months' theoretical and practical training. A written examination from 10 to 1 o'clock was followed by a *viva voce* examination and a clinical examination held in the wards of the Clapham Maternity Hospital. The examiners were three medical women, experts in midwifery, but neither teachers nor lecturers of the institution. Fourteen candidates presented themselves, and of these eleven passed, five obtaining "commendation" for having received above two-thirds of the marks.

### A MATRON'S TESTIMONIAL.

We understand that as the result of "The Testimonial Fund," organised for Mrs. McIntyre, late matron of Sir Julian Goldsmid's Home of Rest for Nurses at Brighton, a cheque for £82 has been handed to her by the honorary treasurer. Mrs. McIntyre has written a letter thanking the contributors, in the course of which she says that it "was with feelings of sincere gratitude that I received from Miss L. Brook-Hunt the testimonial to which you have all so kindly subscribed, and in thanking you all most warmly, I wish at the same time to express my appreciation of the trouble which Miss Brook-Hunt has taken in acting as secretary. In severing my connection with the old home I trust I shall not lose the many friendships so happily begun there. At least, the tangible expression which you have so kindly given of our former links will help me, in entering on my new work, to do it with hope and courage."

### PROGRESS AT LAMBETH INFIRMARY.

The report received by the Lambeth Guardians from the Workhouse and Infirmary Committee, which was considered at their last meeting, shows that, at the recent examination, 11 out of 23 probationers passed in the first division, six in the second division, the remaining six failing to secure the necessary number of marks for a pass. The announcement was made that Dr. Abbott, the examiner, is of opinion that this is the most satisfactory examination he has yet conducted at the infirmary, and the Guardians expressed their high appreciation of the careful training by the medical officer and the matron, to which they attribute, in large measure, the excellent result.

### STOKE-UPON-TRENT UNION HOSPITAL.

The qualifying examination for third-year probationers at Stoke-upon-Trent Union Hospital was recently conducted by Dr. Petgrave Johnson, medical officer of health. The following nurses were successful: A. Faram, M. Caulcott, S. Trotter, L. Faram, and E. Adams.

### DISTRICT NURSES AND INVALID CHILDREN.

In the seventeenth annual report of that admirable organisation, the Invalid Children's Aid Association, allusion is made to the assistance rendered by district nurses. The Committee, says the report, are glad to know that the almoners at the various hospitals and the managers of the schools for defective children are in constant communication with their office or visitors, while to the District Nursing Associations in different parts of London they acknowledge their indebtedness for their readiness to help, either by visiting or by referring cases to the Association. There is a nurse on the Finance Committee of the Association, and we have no doubt that her services are very much valued.

### SHORT ITEMS.

Major Robert Frederick Lee, ex-mayor of Grantham, who died last May, left, according to his will, £100 each to the Grantham Hospital and the Grantham Victoria Nursing Institution, and, in the event of his death without issue, £100 in addition to the latter institution.

### The Nursing Outlook.

"From magnanimity, all fear above;
From nobler recompense, above applause,
Which owes to man's short outlook all its charm."

### THREE INDISPENSABLE QUALITIES.

NURSING is distinctly woman's work. As Dr. Worcester testifies women are peculiarly fitted for the onerous task of patiently and skilfully caring for the patient in faithful obedience to the physician's orders. Ability to care for the helpless is woman's distinctive nature. Nursing is mothering. Grown-up folks when very sick are all babies, and some of us are babies when only slightly ill. In no other employment but nursing can women so well bring into action their highest powers. In so many employments now open to women only their brains are wanted or the use of nimble fingers. Craftsmanship and brainwork are sufficient for men, because they have the incentive to make provision for those near and dear to them. For women it is of the highest importance that they shall find employment in which their hearts as well as their heads and hands shall have full exercise. Nursing of all other employments offers this opportunity and so nursing is the most popular of all pursuits for the majority of women.

For women who aspire to be nurses there are three indispensable qualities. Intelligence, skill, and devotion. The first, in its higher meaning, when nurses first began to be trained and until quite recently, was not always deemed an essential. Intelligence involves education, that is, general knowledge of a relatively high standard. The quality of being intelligent involves the possession of intellect and a power of reasoning and understanding which includes cultivation. It is too seldom recognised that intelligence is not one thing among others in the intelligible world, but the principle in reference to which alone that world exists. It includes acquired knowledge and information, stored up in the mind and so clearly assorted as to be capable of being brought to bear upon the day's work, as and when occasion may arise. Common instinct is sufficient to guard against many harmful things, but intelligence alone can protect us from the intent and deeper agencies of physiological mischief. Intelligence produces tact, and without tact no woman can make a good nurse. Hence intelligence is an indispensable quality in both the professions of medicine and of nursing.

Skill is another essential quality, for it includes reasonableness, powers of discrimination and discernment, understanding and reason. As applied to nursing skill of course involves practical knowledge and ability. It means a readiness and excellence in applying knowledge and scientific methods to practical ends. It also involves expertness and dexterity in the accomplishment of the day's work, combined with a power of action which ministers the maximum of comfort and tenderness in the handling of a patient, and attention to that patient's requirements. All the teaching in the world will not make a skilful nurse, for skill depends not alone on the teacher, but upon the ability and intelligence of the taught.

Devotion includes true sympathy, but in these days sympathy is a word which is often much abused in use. It involves self-sacrifice, and the concentration of one's best gifts, for it entails the definite setting apart of oneself to the deliberate purpose of ministering to the needs of those entrusted to our care. The words of Ruskin may be aptly applied in this connection to the work of a true nurse: "There was still a sadness upon her heart and a depth of devotion in which lay all her strength." How often in the course of history has it been revealed that a devoted woman, surrounded by infinite peril and risks, though frail in body, has been able to show, by her devotion, that her strength, in the discharge of most unpleasant and exacting duties, has far exceeded that of the strongest man. The best of all our nurses have always exhibited the truest devotion, and this attribute, so characteristic of the best of women, must ever be regarded as the brightest jewel in their badge of service for the sick.

Apart altogether from the service which a nurse is able to render to strangers, she is often able to serve her own family and her own friends when ill, so that her combined chances of comforting others and of saving life afford her many heart-satisfying opportunities. In this exercise of all her powers, in this bringing into action her whole strength, she can feel herself fulfilling God's purposes. This alone is real happiness. Dr. Worcester has done good service in making this last point, and we agree with him that the art of nursing is distinctly a Christian art. Certainly the example is still retained of those who feed the hungry and visit the sick in God's name and in the surety that in serving them they are ministering directly unto Him. "But no less beautiful and soul-satisfying is the labour of the modern trained nurse, who, without the religious dress or other outward observances, devotes her life to the service of God in serving His helpless children. In such a nurse's face there shines forth a light which is not of this world." To the workers such words as we have quoted do not always appeal because the work is sometimes done at such high pressure as to be in fact all absorbing. But to the trained observer with knowledge and understanding they are the signs whereby a just appraisement of such work is possible. So the best of personal service is often rendered to others without egoism. And so perhaps the influence of such labours in fact forms the motive power of most vitalising movements amongst peoples all the world over.

### Medical Electricity and Light Treatment.

By Kate Neale, Sister-in-Charge of the Actino-Therapeutic Department, Guy's Hospital.

### I.—INTRODUCTION.

The application of electricity as a method of treatment has of recent years become so much more frequently adopted by medical men that it is by no means unusual for a nurse to be called upon to carry out the instructions of a doctor in this direction. And yet, the facilities she has of acquiring some practical knowledge of this particular side of her profession are far from common. A few of the largest hospitals give a proportion of their nursing staff opportunities to learn the treatment of patients in their electrical departments, but I think it is fairly safe to say that the majority of nurses know little or nothing about the subject. Not that the fault is theirs, for you cannot expect a nurse more or less weary from her duty in the wards to devote the few spare hours of leisure that are hers to the study of a strange subject, and one which has, so far as she can see, no immediate bearing on her work.

But no one I suppose will deny that it is as desirable for a fully trained nurse to possess some proficiency in the use of medical electrical apparatus as it is for her to have experience in massage or fever nursing. Unfortunately her requirements, not only in practical work but also in the matter of text-books on the subject, have not received much attention. There are, of course, many text-books on medical electricity, but they have been written for medical men and require for their understanding a certain knowledge of the principles of magnetism and electricity. Of handbooks for nurses, there is, as far as I know, none, and in this series of articles I shall attempt to supply the deficiency. My intention is to give you information as practical as possible, and to describe only so much of the various forms of apparatus as will help you to understand why you should do this, and why avoid that.

To begin with, you will need to have some idea as to what electricity is. If you rub briskly a glass rod with a piece of silk, both being quite dry, and then hold the rod over some small scraps of paper, or some saw-dust, you will see the particles immediately jump up to the rod. They are attracted by it. The same thing happens if you use sealing-wax or amber in place of the glass, in fact it is this property that amber possesses which has given rise to the word electricity (from "elektron," the Greek for amber). If, instead of rubbing the glass rod with silk, you rub it with a stick of resin and then bring it near a little ball of pith hanging by a silk thread from another piece of glass, you will see the ball at once attracted to the rod. The moment the two touch, however, the ball jumps away again, and the nearer you try to bring the rod the further away does the ball swing. Now hold the stick of resin in the neighbourhood of the pith-ball; it will rush to the resin as vigorously as it receded from the glass.

What then happened when you rubbed rod and resin together? It is supposed that every substance contains an unlimited amount of an "electric fluid," and that on rubbing, this fluid splits up into two sorts, one being called *positive electricity*, the other *negative electricity*. In the experiment just described the positive electricity collects on the glass, the negative on the resin. The pith-ball is attracted by the positive electricity in the glass, and moves towards it, but as soon as they meet the ball receives some of the electricity from the rod, that is, becomes itself charged with positive electricity, and then jumps away again, because any two bodies with the same kind of electricity in them repel each other. Now, when you bring the resin with its negative charge to the ball, the latter moves towards it, since opposite kinds of electricity attract.

You will notice that when the glass is charged you can hold it without its electricity leaking away through your hand; that is to say, glass will not conduct electricity. If you try to electrify a piece of metal, *e.g.*, iron or copper, by rubbing, you will not be able to get any result, because as fast as the electricity is produced it escapes through your hand and body and is lost in the earth; metals, therefore, conduct electricity well. In other words, some substances, such as metals (iron, copper, etc.), are *conductors*; others, such as glass, and also vulcanite, are *non-conductors*. Later on we shall see the importance of this difference.

Although electricity can be produced by friction (*static electricity* it is called), you will find that the majority of the electrical instruments you will have to use derive their power from another source—namely, from some form of battery. This variety is spoken of as "*current electricity*," and before going any further we must try to understand something about batteries, and to become acquainted with a few of the more important terms used in connection with them.

A *battery* is an apparatus for producing current electricity, *i.e.* for producing an electric current. There are many varieties of battery; the one most in favour is called a Leclanché battery or Leclanché cell. It consists of a rod of zinc and a plate of carbon immersed in certain chemicals, and the whole enclosed in a jar, glass or otherwise. The effect of this arrangement is that when the carbon is connected to the zinc by means of a copper wire (copper, we just saw, conducts electricity easily) a current of electricity runs along the wire, and more, it runs always in the same direction—namely, comes out of the cell at the carbon, flows along the wire to the zinc, and then enters the cell again. Special names have been given in connection with this flow. The place where the current comes out is called the *positive pole*, the point where it re-enters the *negative pole*, and in the same way, if you screw one wire to the positive pole and another to the negative, the former is the positive wire, or *anode*, and the latter the negative wire or *kathode*. The screw to which the end of any wire is attached is called a *terminal*. The current always flows from positive to negative, so that when you hear a terminal spoken of as the positive terminal you will know that the current is coming down from the battery to that terminal and, *vice versâ*, the negative terminal has attached to it the wire that takes the current back to the battery.

Different batteries give currents of different strengths, and the more cells used the stronger will be the current; but it is often necessary in treating a patient to use a current of a definite strength, and to this end many forms of apparatus have a dial which shows by means of an index how strong the current is that passes. The dial is called an *Ammeter*—that is, a meter for measuring ampères, an *ampère* being the unit of strength of a current. A current is said to be of so many ampères, according to its strength, so that a current of one ampère will be just double the strength of one of half an ampère. Or, to illustrate the meaning of the word in a different way, when you measure a distance, you say it consists of so many yards; similarly, when you measure a current, you say it consists of so many ampères, and just as you might express very short distances in inches, so very small currents you express in what are called *milliampères*, 1,000 milliampères being equal to one ampère. A current even of one ampère is, under ordinary circumstances, far too strong to pass safely through a human body, and the usual strength employed is only about $\frac{5}{1000}$th of an ampère, or five milliampères, and the dial mentioned above is, therefore, marked off to register milliampères. When some particular forms of treatment are ordered, the doctor will tell you what strength of current he wishes used, and you must be careful to maintain the current at that strength during the whole time of administration. If you make it stronger you might do harm, if weaker, the beneficial results may be lost.

Alterations in the strength of a current are sometimes brought about by increasing or decreasing what is called the *resistance* in the circuit, and a word or two in explanation of this term is necessary. A current, we have seen, will flow along a copper wire, but, though the wire is a conductor, it offers a certain resistance to the flow, thus tending to weaken the current. The longer the wire the greater will be the resistance, and similarly a fine wire offers more resistance than a thick wire. We can, then, easily weaken a current by making it traverse a long coil of fine wire, and you will find that many of the instruments you use will be fitted with "*resistance coils*" or "resistance boxes," so arranged that by some simple movement, such as sliding a terminal in one direction or the other, you can regulate the current at will. Occasionally the resistance is obtained by introducing an electric light in the circuit, the carbon filament of which has a high resistance and so weakens the current.

We are now in a position to understand why some materials are conductors and others non-conductors. A material, the resistance of which is comparatively slight, allows a current to flow easily, and is called a conductor, but in the case of glass we have an example of a non-conductor, since its resistance is so enormous that not even the strongest current can pass. The skin of the body offers a very great resistance to electric currents, but if it is moistened this resistance is decreased. Remember this peculiarity when you come to treat a patient, and always well moisten the skin before you apply the current.

So far I have spoken only of electricity obtained by friction (static) or from a battery (current), but you will find that it is often more convenient to utilise the current supplied for the purpose of electric lighting, and, as a rule, it is this method that is adopted in hospitals. There is, however, no need for you to know how this is effected—it is a question that concerns practical engineers. In private, especially in the country, where no electric light is to be had, medical instruments are used that depend for their electrical supply on batteries. Every instrument maker's catalogue contains illustrations of many such that can be easily carried from place to place, and later on I shall describe a suitable form of apparatus for a private nurse to possess.

Perhaps you have found these introductory remarks rather difficult to understand, especially if you did not previously know anything about electricity, but before going any further it will be wise for you to get a clear idea of what is in these paragraphs, and the meanings of the various terms used, otherwise you will not be able to understand what follows.

### The Nurses' Clinic.

### NURSING AND FEEDING OF CASES OF CHRONIC DIARRHŒA AND VOMITING IN INFANTS. BY A MATRON.

Chronic diarrhœa and vomiting in infants, although not giving rise to the urgent symptoms met with in the acute form, require the greatest care and untiring perseverance and patience.

The little patient may have been submitted to a course of innumerable patent foods, and have been dosed with many prescriptions, generally carried out wrongly. No chance of success had been given to any form of treatment, first one and then another having been discarded, if the first dose was not followed by an instantaneous cure. Consequently, the infant's digestive tract has got into such a state of irritation that every attempt at feeding is met with immediate vomiting.

Innumerable drugs are being tried constantly, some with a fair amount of success, but these do not come within the nurse's province. An initial washing out of the stomach with warm sterile water one pint, and sod. bicarb. one drachm, is liked by most doctors, and if the motions are slimy and offensive, the rectum is treated in the same way, and these two operations are in many cases repeated night and morning for some time. The apparatus in either case consists of a small œsophageal tube or soft india-rubber catheter, a piece of india-rubber tubing about half a yard long, and a glass funnel. The fluid is injected at a temperature of about 100° by raising the funnel slightly above the patient (in the stomach wash), injecting one or two ounces, and then lowering the funnel well below the mattress to empty the stomach. This is repeated until the fluid comes away quite clear, free of mucus and other visible impurities. This washing out is generally done with the child's head hanging over the side of the cot, being careful to have the child well rolled up in a mackintosh and warmly wrapped.

Hot bottles and hypodermic solutions and syringes must be at hand in case of collapse. A gag is rarely necessary, and may be harmful. I have found it convenient and often successful to inject a feed on completing the process of washing out the stomach.

### THE NURSES' CLINIC—*Continued.*

The rectal wash is given with the patient lying on the left side on a mackintosh and towel. The funnel must only be raised a very short distance above the child's body, else the fluid will be expelled before it has travelled any distance. Two or three ounces can be injected and the funnel then lowered.

An initial dose of castor oil may be given by the mouth, so as to try to clear out the digestive tract before starting he feeding.

As a nurse I cannot prescribe any special form of feeding, I can only give an account of the various foods which seemed to me to have been more successful than others during ten years' practical work at a children's hospital in the slums of London, where cases of gastro-enteritis, acute and chronic, abound.

Undoubtedly the one food with which one could hope to be successful with babies under six months of age, is the milk of a wet nurse. We all know that a great number of these poor babies would never have needed to come to the hospital if only their mothers would or could have fed them. Unfortunately in England wet nurses are not yet obtainable in hospitals, but in private, whenever it is possible, mothers cannot be too strongly urged to procure this natural food for their sickly offspring. In America and Germany a praiseworthy effort has been made to keep wet nurses on the premises of infants' hospitals and this attempt has met with most encouraging results.

As a rule it is not much use feeding a chronic case of gastro-enteritis for any length of time on albumen mixture, because the baby has been starved too long through the constant rejection of food.

Slight cases may do well from the start on ordinary milk mixtures: Milk and lime-water, or milk and oatmeal gruel, well diluted. If these mixtures are not retained, peptonised milk or Benger's Food may be used, the last being very much more in favour with the infants than peptonised milk. One of the few artificial foods which I have found a help, is Horlick's Malted Milk. The babies like it, and in some cases it has been entirely satisfactory.

A new preparation, Pegnin, is very well spoken of. It is supplied in the form of a white powder which has to be added to the milk and breaks up the curd into minute particles, thus removing the major factor in irritation.

If all the above foods have been unsuccessful, a mixture, called malt soup, has met with very good results. It is rather tedious to prepare, but I have found it answer in so many cases where everything else has failed, that I have never grudged the trouble. Even if this milk mixture is rejected, yet another method can be tried before finally giving up milk which is the one all-important food for infants. Many of these babies have become so used to vomiting after food that they cannot part with the habit. The thing to do then is to feed them so that they are not aware of being fed, and thus, often, when I was at my wits' ends what to do, I took refuge in the nasal tube, and the patient left off vomiting, and I was soon able to return to the natural way of supplying nourishment.

Of course, everything about the ways of giving the food, said with regard to acute cases, holds good here. Small doses may be tried frequently if larger ones fail; the bottle may be used or the spoon, or even a small medicine glass; the children should be kept absolutely quiet, shut off from excitement and noise, they should be made perfectly comfortable before starting to feed, and left in peace afterwards. In addition a course of open-air treatment, provided it is warm and sunny, may help.

Having tried milk in every form and failed, the last things to turn to are the various beef and other meat juices, either raw or semi-cooked, and given with or without albumen mixture or other diluent. Of these juices the Guy's Hospital Pharmacopœia's beef juice has been most successful with our babies, but while giving this food one must always keep in view the necessity of returning to milk, and reintroduce this carefully at an early date in a very diluted form. For example, if a child takes half an ounce of albumen mixture with 1 drachm of beef juice, one can begin by adding 10 minims of peptonised milk or Benger's food, and if this is not rejected, very gradually increase the amount. Whey or cream mixtures I have not found useful.

Feeding per rectum has been a complete failure in every instance, babies of tender age being apparently quite unable to respond to the introduction of a tube into the rectum otherwise than by expelling the contents.

One thing to keep in mind in feeding these babies is to persevere long enough with one kind of food. A great mistake is made in changing about constantly, and so never giving anything a fair trial.

The same absolute cleanliness, beginning with attention to the mouth—which I mentioned with regard to acute gastro-enteritis—must be practised in the chronic form; frequent baths are comforting and help to keep the withered skin of the poor little infants in good condition. The irritated condition of the skin of the buttocks and round the anus which is often present is best overcome, firstly, by absolutely scrupulous cleanliness, washing the child every time it is found wet, then sponging over with weak boric lotion, drying very well, and afterwards applying either zinc ointment or, if this does not answer, a powder composed of starch, zinc, and calomel.

A piece of warm wool applied over the abdomen with a firm flannel binder seems to relieve pain and restlessness.

Before returning the child to its mother an effort should be made to get it back on ordinary milk; and here, again, I have found Knorr's Hafermehl gruel an excellent diluent in making the milk agree and fattening the baby. The mother ought to receive full instructions about the way of preparing the feeds and looking after the baby generally. I have purposely not mentioned the way in which the milk should be treated before using it for the feeds, because nowadays this is a matter which rests entirely with the medical man. While in this country either sterilised or pasteurised milk is in favour, many Americans will only hear of fresh milk, obtained under "aseptic" precautions, and cooled immediately after the milking process. I append the recipes of the three foods which may not be known to all our readers.

*Malt Soup.*—Two tablespoons of wheat flour are mixed with 12 oz. of fresh cow's milk and strained. In another basin three tablespoons of extract of malt are dissolved in 24 oz. of boiled water at a temperature of 120° Fahr., then two-and-a-half drachms of an 11 per cent. solution of potassium bicarbonate are added. Throw the two preparations together and boil up quickly.

*Oatmeal Gruel.*—Two flat tablespoons of Knorr's Hafermehl (obtainable at Messrs. Allman, 9 Cullum Street, Fenchurch Street, E.C.) are mixed to a smooth paste with some cold water, this is added to a pint of boiling water on the fire, stirred for five minutes and left to simmer for an hour, stirring occasionally.

*Beef Juice.*—One pound of lean beef is finely chopped and added to one pint of water. This mixture is then gently heated (being constantly stirred) until the temperature is 55° C. (130° F.) at which it is kept for 15 minutes. To this is added three ounces of beef-tea and one small teaspoonful of salt. The whole is then strained through a hair sieve or muslin and filtered into the flask in which it is to be kept. If placed in an ice-box the juice will keep sweet for two or three days.

### A Visit to a Berlin Hospital.

BY AN ENGLISH NURSE.

THE Krankenhaus Moabit strikes one on first entering the grounds as being unusual and picturesque, and when I learnt that it was built in 1870 at the time of the cholera outbreak during the Franco-Prussian war I understood why each ward or "station" as they are called, stands alone as did our huts in South Africa. There are 35 such wards, and with their black pith roofs, their quaint overhanging eaves, the walls well covered with a luxuriant growth of creepers of many varieties, they formed a pretty background to the many groups of patients who in clean linen suits sat about in the bright sunshine. The operating block is most complete. There are three large operating rooms, the largest having three tables. In addition there is an operating room specially for eyes, ears, noses, and teeth. In none are seats provided for students or other visitors. There are two dark rooms, one for the Röntgen ray apparatus and one for photographic developments. Rooms are provided for the examination of patients before admission into some of the special wards; for the examination of specimens, etc., and sitting-rooms and bathrooms for the doctors. The equipment of the operating rooms is much the same as it is in most other hospitals I have seen, though there are a few ingenious contrivances which I was not familar with, such as an arrangement for forcing a narrow volume of boiling water through a silver catheter or similar instrument, with such force as I think would defy the most hardy microbe to withstand.

### THE OTHER BLOCKS.

From the operating block we passed along, just glancing at the shops of the locksmith, the joiner, the painter, and the mattress-maker, till we came to the disinfecting block, where there are huge iron safes in which are baked the mattresses, also the patients' clothes taken from them on admission. The laundry block is a large red brick building; the upper floors contain the rooms in which the 28 laundresses live, and they looked bright and homelike with their little balconies and windowsills gay with flowering plants. Then came the kitchen block, the most wonderful of all, with its vast white-tiled kitchen, containing a dozen large boilers, in any one of which four ordinary people might hide themselves. There is a butcher's room where the many different kinds of sausages are made. In the cold storage room, with its beautiful shiny white tiles, and out of which open two large ice chambers, also white-tiled, hang many whole carcases. There is the cool dairy, round the sides of which are arranged, on the floor, large marble baths. In these the fish, vegetables, etc., were prepared, each different thing having its own special bath. The meals are conveyed to the wards in thermophors on transport wagons. Upstairs again the score or more of kitchen-maids are housed. They have large, cheerful, well-furnished bedrooms. There are bathrooms with nickel-plated baths and a wash-tub for washing their garments at any time, and another room round the sides of which are arranged white earthenware wash basins, mirrors, and an apparatus for heating curling tongs, such as one sees in a hair-dressing establishment. At first I thought that it seemed startlingly considerate of their small vanities, but it is probably only a wise precaution against methylated spirit lamps and the consequent dangers of fire. In this room also is provided every convenience for the ironing of any garment.

### THE NURSES' HOME AND STAFF.

The Nurses' Home struck me as very dreary in its severely clean, tidy, almost prison-like appearance, not gay and bright and homelike such as most of ours are nowadays. The nurses or sisters, as they are all called, were quite delightful and charming, and gave me coffee and cake in true hospital fashion in the little sitting-room off the ward. They all wear aprons which completely envelop them, with long sleeves, so that the Galatea stripe dress beneath is never seen. The junior sisters wear Sister Dora caps without strings, while the senior or head ones have an arrangement of spotted net and lace. They enter the hospital first for a year's probation, making a deposit of 200 marks, which is returned at the end of four years if they stay, otherwise it is forfeited. After the year of probation they start at a salary equivalent to 10s. a month. In the third year this is increased every third month by one mark. The head sisters begin with 45 marks a month, 55 marks being the maximum. The sisters have no lectures and no examinations, there are 70 of them, but they nurse only in the women's and children's wards, the men having male attendants.

### WARDS AND LAVATORIES.

The wards are much like other hospital wards, but I was much impressed by the lavatories with the large boilers provided for the boiling of all bed-pans and urinals, and also by the large tanks of disinfectant for receiving the soiled linen. I was sorry for the night sister in one ward where the bedside tables had lead tops which shone like silver, and one of whose many duties it was to keep them so! In the children's ward the bath- and dining-rooms are furnished with miniature tables, chairs, and baths, kindergarten fashion. The children's wards are visited three times a week by a kindergarten teacher who interests and amuses those who are fit for it. I left the sisters, assuring them of the hospitality of English sisters, and of the hearty welcome they would certainly receive should they at any time wish to see over an English hospital.

### Some Experiences of a District Midwife.

WHAT my feelings were when, one never-to-be-forgotten morning, I started on a fresh episode in my nursing career, no one but myself knows. I had just concluded part of my maternity training in the wards of a large lying-in hospital and was now to profit by that experience under very different surroundings.

I was presented with a bag—a thing I never loved—and given a list of names and streets, which might have been at Jericho for aught I knew. In answer to my anxious inquiries as to what I was supposed to do, Sister cheerfully remarked, "Oh, you'll be all right, nurse; do the same as you do in hospital." This sounded comforting and off I started. After inquiring of every policeman and losing myself a dozen times in many tiresome and tortuous turnings, I found my first patient. According to all text books a nurse should always greet her patient cheerfully. In my anxiety to carry out this excellent rule I had not noticed that the family coal-box and cradle combined had been placed beside the bed. My patient's startled exclamation made me realise that a tragedy had only just been averted. Amongst Sister's earnest instructions was one which all district nurses will do well to carry out—always put your cloak on a chair or a table. I looked round for a chair, and discovered a few remains carefully concealed under numerous garments, all of various intensities of grime. Having tightly and firmly rolled my cloak until it resembled a hedgehog, I proceeded to take pulse

SOME EXPERIENCES OF A DISTRICT MIDWIFE—*Continued.*

and temperature. "Mother," having been an inmate of some "'orspital," was quite *au fait*, and did not proceed to chew the thermometer, as has been known to happen. Over the pulse, however, we were not quite so happy. One hand only was I allowed to grasp, because the last nurse preferred it. The daily washing was another delicate subject. "The lady upstairs thought you was so busy, nuss, so did me afore you came;" but nurse was firm, and proceeded to business. The difficulties of the situation were better revealed every minute. "Do as you do in hospital;" well, I can't confess to finding much resemblance, but fortunately the results seem satisfactory.

The bathing of the baby is an event of great excitement to all the children and half the women in the house. Nurse undergoes friendly criticism, with an undercurrent of feeling that "she is only larnin'," and not to be compared with the qualified ladies who usually attend these festivities.

There are certain theories connected with this work, which, strange to say, are not taught in hospital, but which are handed down from generation to generation, and which no amount of Board School, sanitary inspectors, nor trained nurses will ever eradicate. One large subject is "Infant Feeding." After a week's experience it did not disturb me in the least to find a four hours old baby having a good feed of oatmeal porridge because she cried. "Little dear," as Granny casually remarked. "All her brothers and sisters 'ad it." An inquiry as to the number of children is usually met with the number of funerals the family has indulged in. Mourning cards, neatly framed, form the chief ornament in many an otherwise cheerful house.

The lady in attendance on these occasions is often more a hindrance than a help. One was a great trial; at the critical moment she would exhort the patient in language which is usually met with at prayer meetings of the fervid order, when the orator wishes to bring the point home in the plainest way.

A few interesting facts are worth noting. Most women at the earliest possible minute gaily get up and go on with their daily occupation. It is easy to blame them, but with half-a-dozen other babies and no friendly neighbour, what is to be done? A child is posted at the top of the street to watch for nurse and give due warning. On her approach, mother hops into bed, and should nurse omit to turn up the bedclothes and look for feet adorned with boots, all may yet go well. But nothing will induce any woman to get up on the ninth day. "Oh, no, nuss, yer bones joins together that day," and nurse is looked upon as the most ignorant being on the face of the earth. One learns much as to the various causes of different things. Poor little Billy, who, to nurse's experienced eye, is as rickety as he can be, can't walk because he is cutting his teeth, and they "catches him across the loins." If a baby chokes whilst feeding, "the milk has a bone in it." Rabbit's, or, better still, hare's brains are considered excellent diet for an infant. A baby born with rather more hair than is usual, one is immediately told "Of course 'Liza expected it, 'cos she 'as 'ad 'eartburn that awful."

One of the funniest things was solemnly told my fellow nurse. At the usual time the patient went up to the hospital to be interviewed. On her return she told her sympathetic friends and relations that she had undergone great suffering, having had "instruments." Further inquiry elicited the fact that the instruments referred to were the "callipers," used for measuring the pelvis. Taking an "off-day" duty, I heard of a terrible operation the day before, which proved to be nothing more formidable than a douche.

We had an amusing adventure one night. We had been to a case in the afternoon and were paying our second visit. Marching down the area steps, we knocked at the door and went straight in to the case as we always did. Instead of our patient, to our amazement we found a place full of men, smoking and drinking. We had mistaken the street, and were in a lodging-house. My companion fled precipitately, whilst I, murmuring an apology to the gentlemen assembled, also hastily retired, inwardly convulsed, as the nurses with the baskets and their errand are both well known.

Who does not know the small boy, who, with a broad grin, yells, "Got a biby in y'er basket, nuss," this being a fallacy they think well to uphold.

Poor babies. One sees the most beautiful children, and leaves them at the end of 10 days, knowing, alas! that in a few months' time many of them will add to the ever-increasing crowd who flock to the out-patient departments of our hospitals. It is rather depressing sometimes, but one cannot help but admire the kind-heartedness and philosophy of these people, careless and ne'er-do-weels though they may be.

### The Exclusion of Light and Noise.

### EXAMINATION QUESTION FOR NURSES.

The question was as follows:—In nursing a patient with brain disease or injury, from whom it is necessary to keep all light and noise, how would you arrange the lamp, and light from the fire at night, and how would you avoid any noise in keeping up the fire?

### First Prize.

Procure two screens or a clothes-horse, three-fold if possible. Remove all light articles from the mantelshelf. If clothes-horses are used cover with a dark table-cover or counterpane, and place one on either side of the fireplace well outside the fender. Next place some heavy material—such as a dark winter curtain—over the mantelshelf, allowing an end to hang over each screen. This forms a tent and prevents the light falling on the ceiling. If there are nails in the wall the curtain may be placed a little above the mantelshelf. Then place the screens so as to exclude any light but not heat. Place the lamp on a table behind the screen farthest from the patient's bed, covered with a green shade. Nice little shades for tops of lamps are easily procured. If nice high screens are used the lamp can be placed inside the tent. The screens can be arranged to exclude light in this way, whatever the position of the bed, but it is sometimes more convenient to join the two screens and have an opening at the side of fireplace. The cover for the top is important.

Pull out the fender and place sand on the hearthstone to prevent the noise of cinders falling. Have small lumps of coal placed in the scuttle, each lump separately wrapped in old rag—paper is, as a rule, noisy unless very soft. Have an old pair of gloves and remove the rag or paper before placing on the fire, otherwise it will become clogged. Use stout piece of stick for a poker. "Simplex."

### Second Prize.

In nursing a case of brain disease or injury, from whom all light and noise must be excluded, I should suggest that, first of all, the lamp should be put on a small table, taking care to have a tall screen between it and the patient. Then get as much thick dark brown, or preferably dark green, paper as would wrap round the lamp globe. Pin this round it, only allowing a small chink on the side furthest from the patient for a ray of light to penetrate for one's work or notes. If the patient were in such a position as to be affected by the light from the fire a draw-sheet or something similar could be thrown over a towel-rail, extending one end of the material on to the mantelshelf, where it could be kept in position by books or some such weight. This is easily arranged in such a way as to prevent the light from falling on the patient, and also avoids the flicker on the ceiling.

To keep up a fire without noise the coalscuttles should be filled with small lumps of coal, so that each piece can be lifted on to the fire separately, For this purpose one must be previously provided with a large old glove for the right hand. If it is found impossible to be always provided with lumps of coal, one should have the small coal put into light paper bags or small paper parcels, instead of being loosely put into the scuttles. These parcels of coal, including the paper, can be placed as required on the fire, which is quite a quiet method. When poking the fire or raking out the ashes a piece of stick is more silent than an ordinary poker, as it does not make such a noise if it comes in contact with the bars of the grate. "K. J. S."

THE PRIZE-WINNERS.

"Simplex" gains the first prize because she mentions the three most important things to be considered: how to exclude too much light from the bed, how to make up and keep up the fire noiselessly, and (which is most desirable), how to keep the fire from flickering on the ceiling, a form of annoyance to which patients with brain irritation are especially sensitive.

"K. J. S." comes out second with a good paper, but a drawsheet, or indeed anything white is unsuitable to hang from the mantelshelf; it should be a fairly thick material and of a dark shade. She omits to mention the very necessary precaution of putting sand or earth on the hearthstone to deaden the noise of falling cinders.

THE PAPERS AS A WHOLE.

The papers are good this time, but they still show a want of concentration of thought. For instance, many writers spend time in describing how they should have the patient at the back of the house, lay down straw outside, arrange dark blinds, etc. . . . If they had read the question carefully they would see that they are only asked for information on two points—how to avoid light at night and how to keep up the fire noiselessly.

HONOURABLE MENTION.

This is gained by "Poppea," "Reading," "Harriett," and "Mat."

CLOSE OF THE EXAMINATIONS UNTIL OCTOBER.

This is the last question until the autumn. Due notice will be given of the resumption of the questions.

THE EXAMINER.

### Nursing the Schoolboy.

BY THE MATRON OF DENSTONE COLLEGE SANATORIUM.

(*Continued from page* 227.)

MANY rules which are necessary and admirable in a large hospital, where the nursing is divided between many nurses, are not required when the entire charge rests with one nurse, who never need leave her patient unless she wishes to do so.

But the remarks I am about to make will appeal more to the matron not technically trained in nursing than to the trained nurse. I have no desire, neither would I presume, to teach those of my own profession. I have no doubt that every trained nurse, when she leaves hospital and works independently on her own responsibility, adds her own ideas to what she has been taught, and if she has been a patient herself during a serious illness, many little points she can also look at from a patient's point of view; and I think it follows that she will find details, needful in a hospital, can without any bad effects to her patient, be discarded in the more homelike nursing of a school sanatorium.

Boys are naturally very reserved when ill, and excepting the "shammers," who are easily detected, they do not volunteer much information as regards their symptoms. Temperature often means nothing; a highly-strung, nervous boy will have a temperature of 103, and even more, with an ordinary headache, which an aperient and a night's rest will entirely cure.

SHAMMING.

It is always safe to give a boy an aperient when he makes a complaint, and I invariably seize the opportunity, even if there are no definite symptoms of its being required, as in nine cases out of ten the minor ailments of a boy are due to "tuck." Never leave a boy, who looks well, with a thermometer in his mouth, even for an instant. Boys have wonderful methods of causing the mercury to rise. For instance, if they are near the fire or a lamp they bring the thermometer in contact with the heat, or they fill their mouths with hot water just before they know their temperatures will be taken. Sometimes they succeed to such an alarming extent that the fraud is immediately detected; but if the boy is clever enough to send the mercury up only one or two degrees, although there are no other symptoms of illness, he is generally given the benefit of the doubt, and so escapes school.

Boys have various reasons for "shamming." With some a dislike for work is chronic, but even with workers there occasionally comes a time when to escape school seems to them advisable. It may be a specially difficult lesson that morning which they have failed to prepare satisfactorily, or "impots" may be due which are still unwritten, and a day in the "San." will tide over the difficulty. Never accuse a boy of "shamming" unless you are certain of the fact; boys are most sensitive on this point if they really feel ill, and it is better to give the boys the benefit of the doubt than to send one into school who is not fit to be there. It might not only lead to a serious illness, but would do much harm to the school and weaken the confidence which the authorities, the boys themselves, and the parents ought to feel in the matron. I never encourage "coddling" with an ordinary cold when there is no temperature. I find that a simple stock medicine, made up, of course, by the school doctor, a hot drink at bedtime, and a cup of good soup at the "quarter break" for a few days, is all that is necessary, and the boy will be stronger, both physically and morally, for not having given in.

LIABILITY TO PNEUMONIA.

I find that among schoolboys the most common ailment of an acute nature to which they are liable is pneumonia.

In most of the cases which have come under my care the disease has attacked the boy suddenly, and he has not complained until it has fully developed. While waiting for the doctor I always apply to the affected side a poultice made of one-third mustard and two-thirds linseed meal. I keep this on for two hours, or as long as the boy can bear it; when I take it off I apply one of linseed meal only, which is changed every four or six hours according to the doctor's wishes.

Until after the crisis I give my patient very little milk, not more than one pint in the 24 hours, and that freely diluted with barley water. I also give lemonade made from the juice of the lemon only, and as much cold water in reason as he desires. I rely for nourishment on Brand's Essence and Valentine's Meat Juice, giving two teaspoonfuls of the former, melted, and one teaspoonful of the latter, in a little warm water, every three or four hours. I find that as long as the temperature keeps between 103° and 105°—which it generally

### NURSING THE SCHOOLBOY—*Continued.*

does for the first four or five days—the less food I give in bulk the better for the patient.

Milk, soda-water, home-made beef tea, all, in my opinion, cause more or less flatulence and discomfort. As a rule one bottle of Valentine's and two tins of Brand's Essence are sufficient until the crisis, after which they are not required. The crisis generally occurs between midnight and 4 A.M. on the fifth or sixth day. As soon as I feel the skin on the face slightly moist I renew the poultice, refill the hot-water bags, put on an extra blanket, and give two or three teaspoonfuls of brandy in warm water, repeating it in an hour or two if necessary; but if the pulse is good I do not give a second dose. My experience is that if the temperature does not become sub-normal, but falls gradually, I have practically no exhausting symptoms to contend with, and the patient makes a rapid recovery.

If my patient has passed the crisis safely during the night, I give him for his breakfast a cup of hot milk with bread crumbled into it, at midday a little good soup with dry toast; the same for tea and supper, in little larger quantities. The next two or three days he has fish or chicken, and passes gradually on to ordinary diet.

I have never had a relapse, and my patients have been able to get up within a fortnight from the commencement of their illness; but I never leave them, night or day, until after the crisis, and that I think is one of the advantages of home nursing.

### Other Ailments.

Of less severe illnesses, bilious attacks and a mild form of influenza, induced most probably by a chill, are the most common. For the former, a day in bed with semi-starvation, and the next day feeding up with plain wholesome food, is all that is necessary, and the boy is generally back in school on the third day. For the latter complaint, two or three days in bed, and then a tonic and a little extra "feeding up" for a week, is the usual treatment.

Never, as I said before, be alarmed at a boy's temperature as long as his pulse and respiration keep good and there is no localised pain; I have had scores of boys whose temperatures, even in these short illnesses, have been 103° and 104° the first day or two.

For diarrhœa I always give a dose of castor-oil. This is a favourite disease with "shammers" until they know the remedy, and even then some will submit to it rather than face what they know awaits them in school. In this school, which is most healthily situated, tonsillitis is almost unknown, excepting with a few boys whose tonsils need attention.

*(To be concluded.)*

### Central Midwives Board.

### A CONFERENCE ON THE QUESTION OF EXAMINATIONS.

A meeting of the Central Midwives Board was held on Thursday of last week. There were present: the Chairman (Dr. Champneys), Dr. Dakin, Miss R. Paget, Sir William Sinclair, Miss Wilson, and Mr. Parker Young.

The first business was the adjourned consideration of a letter from the clerk of the Breconshire County Council, transmitting a report and scheme adopted by the Council for the administration of the Midwives Act, and inviting the criticism of the Board. The secretary was instructed to merely acknowledge the letter, with thanks for the information sent.

Memorials from Queen Victoria's Jubilee Institute, and the Executive Committee of the Association for Promoting the Training and Supply of Midwives, and a letter from the Warden and Matron of the Salvation Army Maternity Hospital protesting against the recent decision of the Board to hold four examinations in the year instead of three, and asking the Board to reconsider its decision, were then considered.

The Chairman said he thought that the best solution of the difficulty would be to have a conference early in the autumn, for without hearing the views of all the people concerned they could not come to a good understanding. There was considerable divergence of opinion on this point among the members of the Board. Sir William Sinclair wished to suspend the previous resolution of the Board. The secretary explained that the October examination was fixed, and that he was already being asked about the January examination. Miss Paget and Dr. Dakin, speaking respectively for the Queen Victoria Jubilee Institute and the lying-in hospitals, while anxious for the Board to reconsider its decision, were sure that it would be quite impossible to alter the present arrangements immediately, as it would upset the work of training entirely. They therefore supported the Chairman's suggestion of a conference. Sir William Sinclair then pressed for the conference to be held in July or August, and after considerable further discussion it was fixed for Saturday, July 29th, at 2.30.

Letters were next read from Dr. A. W. Aldrich, complaining that he had been called in by a certified widwife and been paid only half a guinea, while his usual charge was one guinea; and from Caroline Edwards, a certified midwife, stating that the doctors in a certain district had banded themselves together not to take less than two guineas if called in by a midwife, and pointing out the difficulties and hardships resulting. The Board decided that the matter was beyond their jurisdiction, and instructed the secretary to reply that they had not fixed any scale of fees.

It was decided merely to acknowledge a letter from the clerk to the West Riding Sanitary Committee forwarding copy of a resolution passed by that body as to the training of midwives.

The report of the Examination Committee having been accepted, Miss Paget gave notice that she wished to move at the next meeting that a copy of Clause E be supplied to every midwife when she obtained her certificate.

The Chairman then gave the verbal report of the Standing Committee which met the same afternoon. The committee had decided to refuse the application of the West Derby Union Walton Workhouse for approval as a training-school, but the Board reversed this decision. The following were also approved as training-schools:—The Birkenhead Ladies' Charitable Institution and Maternity Hospital, the Cardiff and District Branch of the Queen Victoria Jubilee Nurses' Institute, and the Jessop Hospital for Women, Sheffield.

The next ordinary meeting of the Board will be held on July 27th, and a special meeting to hear charges against midwives on July 25th at 2.30.

### A Book and its Story.

NEW MEN AND OLD ACRES.*

MISS DEANE'S new novel is the story of a business man who buys an old estate and settles down amid the traditions of a family whose past doings had given the place an uncanny reputation. The family ghost is the apparition of a lady who was drowned in a pond in the park, and invisible hands touch the keys of an old harpsichord in a gallery in the house without any apparent result except to warn the present owner, George Donnithorne, that there were ancestral spirits still hovering over a domain to which his claim was too new to be taken seriously by them as a reason for retiring altogether. The story opens with the arrival of Donnithorne at Fraggarts, immediately after the purchase of the place, and with a sketch of himself.

"Fraggarts was sold at last. It had been in the market for some time, and, in spite of its being in a beautiful part of Surrey, had hung upon its impecunious owner's hands until George Donnithorne retired from business and sought a permanent home not too far from London. . . . He was a a man to whom the excitement of gambling did not appeal; he had other interests. His business, although he had a clear and acute head for it, never became an octopus sucking out the substance of the brain and the life of the heart. He was rich, and he did not desire one of those unwieldy fortunes whose mere management consumes a man's time and energy. He had been brought up in the country, had been at one of the great public schools, and to Cambridge. Had not circumstances dwarfed talents into tastes he might have done remarkably well in one of the professions, or become a writer. As it was, books took the place of cards in his spare hours, and his holidays were spent in remote travels or in sport. But he was not an unsociable man. He made new friends and never lost old ones. . . . He was still a bachelor, though he had passed his fiftieth birthday by a few short years . . . and he was a good-looking man, with a light-built figure rather above the medium height, good features, a clean complexion, and a calm, clear look that was an excellent index to his character." Donnithorne, as he is thus described, is lacking in none of the elements which go to form an interesting personality. But, in spite of this, he fails to grip the reader. Of his excellence there can be no doubt, but he is dull. Not a man of sentiment, he had yet in earlier days cherished an unrequited affection for a certain young lady named Isabel, who, not having penetration enough to discover the sterling worth of the lover behind the screen of obscuring self-consciousness which hid his real worth, went off to India and married someone else. When the later chapter of George Donnithorne's life commences with the purchase of the Sanfrey estate, known as Fraggart's, he was not entirely without some one who had claims on him; for on the death of Isabel he had taken her only son into partnership, thus securing his future and enabling him to marry. The young Carberrys stood in friendly relation to the solitary bachelor, and in Roger, his adopted son, and his wife, Jeanette, he found all that could, in the circumstances, be desired.

The late owner of Fraggarts was the descendant of a race of gamblers, according to the fashion of their day, and in his own person presents a repulsive picture of the worst type of unprincipled adventurer. He does not figure long in the story. Having got rid of the estate, he disappears suddenly, leaving his daughter in possession of the cottage in the grounds and some family pearls as the only available asset on which to count for the future.

"The squat little cottage stood in the midst of a small plot of garden within tarred palings. There was a squalid air about it which no effort on the part of the Sanfreys could ever dissipate. . . . A rustic arch over the entrance door seemed to be breaking down, and the last touch was given by dogs' platters strewed negligently in front of the house, with an old bone here and there, on the weedy path." If the exterior was unattractive the interior revealed in its tasteless decorations a revelation of frivolous personality in the occupant herself. "The little square room was ill-lit and shabby to begin with; but what rendered it hideous were poor Georgina's efforts at decoration, with which she was perfectly satisfied. . . . Bows of pink muslin in every direction . . . . shapeless heaps of pots and baskets with ferns and dried grass . . . . trails of ivy, among which were birds' nests, bulrushes, and damp moss."

George Donnithorne, in becoming the purchaser of Fraggarts, we read, "had no idea that the last surviving Sanfrey would form, as it were, part of the bargain, and he was conscious of a sudden wish that he had never seen the place."

Georgina Sanfrey's first interview with him was as follows. He was driving home one afternoon when his eye caught a flying apparition in the distance; he was surprised to see a hand waved towards him, and as he drew near he saw that it was Miss Sanfrey herself. "He saw a small pale face with eyes that looked dark in it, a tumbled pile of corn-coloured hair, a slender figure, certainly not over five feet two. . . . Dark eyes gave a peculiar effect in combination with that light hair. . . . Her eyes were pathetic, but not with human pathos; he had often seen a dog look up into his face with that look in eyes of an indistinct beryl colour. 'So you have come,' said Miss Sanfrey in nonchalant tones, and with the directness of a child, though she must have been seventeen or eighteen. 'And Fraggarts belongs to you simply because you have bought it; but I warn you, I was here first, and you shall not turn me out. . . . The cottage is the only thing that does not belong to you. . . . I wanted to see you. I'm glad you have not brought any girls to Fraggarts; they are so disagreeable. She gave a curt nod, and ran across the park again. He looked after her as he drove on, wondering.' "

"The incident gave a turn to his thoughts which had been growing too melancholy. . . . After all what had he to do with this place? He had no roots in the soil, no fibres reaching far back into the past. That queer little girl whose pathetic eyes haunted him was part and parcel of Fraggarts —but he was a stranger—he was not sure that he was not an intruder. . . . He was sorry for the daughter of Mr. Sanfrey, and compassionate over a spoilt young life, but she was in no way an attractive personality. He was not even sure that she was unaffected, but rather suspected that in a rougher way she showed the same taint of unreality as her father—an insidious disease that he abhorred."

How poor George Donnithorne acted in the difficult circumstances in which he is placed readers must see in the book itself. The characterisation is consistent throughout; but, as a whole, "The Little Neighbour" is not up to the standard of a previous charming story, "Treasure and Heart," by the same author.

* "The Little Neighbour." By Mary Deane. John Murray. 6s.

### Everybody's Opinion.

[Correspondence on all subjects is invited, but we cannot in any way be responsible for the opinions expressed by our correspondents. No communication can be entertained if the name and address of the correspondent are not given as a guarantee of good faith, but not necessarily for publication. All correspondents should write on one side of the paper only.]

### LEICESTER WORKHOUSE INFIRMARY.

Mr. A. B. Chapman, master of Shoreham Workhouse, writes: I see in your announcement of the appointment of Miss A. B. Clarke as matron of the Leicester Infirmary that she is described as having been matron of the infirmary here, but, as you will see by the "list of officers" enclosed (taken from the "Guardians' Year-Book"), her post here was that of superintendent nurse only. The infirmary here is not under separate administration, but is managed by the workhouse master and matron—that is, my wife and myself.

### A GRIEVANCE.

"Male Nurse" writes: Whenever I am in want of a case I always advertise in the Nursing Section of The Hospital, as I find it the best and most reliable paper for such advertisements, and all the cases I have had for 10 years have been got through it. But may I be allowed space to air one real grievance; though it is not the fault of The Hospital, but of some of the readers, who for curiosity or other selfish motives answer the advertisements of nurses, and put them to a deal of trouble and waste of time, when they have no intention of engaging them? I know of a lady in the West End who often gets nurses to call for interviews, but never requires one. And I have had a postcard several times asking me to call at an address, only to find that it does not exist.

### COLONIAL SISTERS AND AMATEURS.

The Matron of the Government Civil Hospital, Hong Kong writes under date of June 2nd, 1905: I was much surprised and annoyed to read in your valuable paper of April 29th (which arrived by yesterday's mail), that the sisters of the Government Civil Hospital objected so strongly to teaching a few ladies a little practical nursing, as almost to amount to a strike! I should be much obliged if you would deny this statement most emphatically on behalf of the sisters. They are naturally very disgusted and indignant about it. The doctors gave a course of lectures on "First-aid Nursing" to some of the ladies of the colony; it was then thought it might be useful if they could learn a little medical work in the wards, so as to be of some little use in an emergency or epidemic, as it is most difficult to get outside help of any description at such times, as the few ladies who come (and they are very few) only come for one hour once a month at a set time. It is scarcely likely any sister would trouble to make a fuss about it. If it is not a breach of confidence, I should be much obliged if you would tell me where you got this information from.

[We do not under any circumstances disclose the sources of information which is sent to us from private correspondents, but in this instance, the statement on which we commented was made in the columns of the leading Hongkong newspaper.—Ed. The Hospital.]

### THE NEED FOR REGISTRATION OF NURSING HOMES.

"A Victim" writes: I was rather amused at "Fair Play's" championship of private co-operation institutions, and glad to find that her experience, and that of "all the nurses she knows," has been so much happier than my own. Certainly the principal of an institution of this kind would expect to make "something towards rent, rates, taxes, wear and tear of furniture, etc.," and would have ample opportunity of doing so at the institution to which I alluded, seeing that there were about 24 nurses on the staff, each paying her 4s. a week commission whether they were earning anything or not! Again, "Fair Play" is under a grave misapprehension as to my "not getting work through the home because I was engaged on my own cases." I before stated that I, and other nurses, had often waited weeks in the home hoping for work, and eventually, in despair, took any work we could get of our own. I do not think either that there could be any question of the nurses "picking and choosing" cases, seeing that the cases offered were so few and far between. A nurse has the right also, in my opinion, to refuse an unsuitable case—as, for instance, when she is offered a dipsomaniac case with no doctor in attendance. I cannot see what objection there can be to registration and inspection by any home worked on a fair co-operative principle. There are doubtless some private co-operations where the comfort and interests of the nurses are considered. They certainly were not considered in the institution to which I belonged for 12 months; neither does it speak well for that institution that, out of a staff of 24 nurses, 14 "were malcontents," and left the institution before the end of my time.

### Appointments.

[No charge is made for announcements under this head, and we are always glad to receive and publish appointments. The information, to insure accuracy, should be sent from the nurses themselves, and we cannot undertake to correct official announcements which may happen to be inaccurate. It is essential that in all cases the school of training should be given.]

Guildford, Godalming and Woking Joint Hospital.—Miss Katherine Emma Mayhew has been appointed charge nurse. She was trained at the Borough Isolation Hospital, Leicester, and has since been assistant nurse at the Eastern Hospital, London, staff nurse at Hastings Sanatorium, staff nurse at Leyton Hospital, and first assistant nurse at the Fountain Hospital, Tooting, London.

Hatton, Ceylon.—Miss M. E. Jolly has been appointed sister by the Colonial Nursing Association. She was trained at the General Hospital, Ipswich, and has since been nurse at the Victoria Park Chest Hospital and sister on the staff of the Registered Nurses' Society. She holds the certificate of the Central Midwives Board and a certificate for massage.

Jenny Lind Hospital for Children, Norwich.—Miss Florence K. Pratt has been appointed lady superintendent. She was trained at Leeds General Infirmary, and has since been sister at the Victoria Hospital, Chelsea.

Leek Workhouse Infirmary.—Miss Margaret E. Okey has been appointed superintendent nurse. She was trained at Birmingham Infirmary, and has since been superintendent nurse at Cheltenham Union Infirmary, sister at Birmingham Infirmary, and sister at Worcester General Infirmary.

Nottingham Children's Hospital.—Miss F. M. Payne has been appointed staff nurse. She was trained at Birmingham General Hospital.

Plymouth Workhouse Hospital.—Miss Josephine Bird has been appointed sister-in-charge of the female wards. She was trained at Jervis Street Hospital, Dublin, and the Coombe Lying-in Hospital, Dublin, and has since been night superintendent at Jervis Street Hospital and Queen's nurse in Ireland. She holds the certificate of the Central Midwives Board.

Union Infirmary, Whiston, Prescot.—Miss Kate Johnson has been appointed sister. She was trained at Bradford Union Hospital. She has since been sister at Sheffield, and has done private nursing on the staff of the English Nursing Institute, San Remo. She holds the certificate of the Central Midwives Board.

### Death in Our Ranks.

The death took place on June 25, at Eastbourne, of Miss Ellen Hobby, for many years on the staff of the Nurses' Institute, 78 Lansdowne Road, Croydon.

The death of Nurse Hilda Love, of Stroud Hospital, took place suddenly on June 30 at Clifton, after only a few days' illness, and while she was on her holidays. She was trained at Barnstaple and Bedford, was three years staff nurse at the Birmingham General Hospital, and for the last 18 months charge nurse of the theatre and women's wards at Stroud Hospital.

### Novelties for Nurses.

(By Our Shopping Correspondent.)

### NURSES' APPLIANCES AT MESSRS. BAILEY'S.

Nurses come and nurses go, but their wants seem not only to go on for ever, but to increase as they go. This occurred to me other day when I was looking at the up-to-date appliances at Messrs. Bailey's, Oxford Street. Perhaps a description of some of the things I saw would be useful to district nurses and midwives.

First, then, I saw a bag made of waterproof cloth. Inside a fitting lining, made to button on and take out for washing purposes; spare linings can be obtained, or would be little trouble for a nurse to make herself. The bag is a convenient size to carry for walking or bicycling purposes, and is as light as possible. I was astonished when I lifted it. It is fitted with the following: Clinical thermometer, pulse glass, sterilisable enema syringe, scissors, iodoform powder dredger, unglazed thread, four bottles, two of which are blue for poison, a pot for carbolised vaseline or ointment, carbolic soap, and a vaginal glass tube. Fitted thus the bag may be had for the moderate sum of 10s.; empty, 4s. 9d.

The next I looked at was black, made of cowhide, also with removable linings. It contained in addition to the first, five stoppered bottles, one blue, two ointment pots, a medicine glass, and minim measure in a case, a bath thermometer (also in a case), absorbent wool in a neat waterproof case, and soap and nail-brush (also in a case). This costs 17s. 6d. complete, 8s. 6d. empty. Between these and the bag I am about to mention, I saw several others varying in price, with improvements in the quality or more fittings accordingly; for instance, one make has an outside pocket, useful for many things if not absolutely necessary.

Yet another, made of cowhide, is square-shaped, and opens wide. It is fitted with an indiarubber catheter, sterilisable "holdfast" enema syringe, rotunda douche, glass vaginal tube, sterilised ligature, tow, wool, soap-case, scissors, medicine glass, and minim measure in a case, kidney-shaped tray, bath thermometer, eye-dropper, safety-pins, three pots, and six stoppered bottles. It costs 32s. 6d. complete, 14s. 6d. empty. The enema syringe is a great improvement on the less modern kind; it guards the end of the syringe which is placed in the bowl from slipping, which not only leaves the nurse one hand free, but prevents air being injected.

Later I was shown an ingenious tin steriliser with folding feet, containing many things; the whole shuts up into a convenient size, and there is a washable outside case, into which it fits, with straps and handles for carrying purposes. Its contents consist of rotunda syphon douche with holdfast end, tin douche can, enema syringe sterilisable, a neat small nickel box, with division for soap and nail-brush. A compact tin case deserves notice: it holds four bottles, an eye-drop bottle, dredge, ligatures, glass reel, and medicine tumbler, all of which are enclosed in the case, occupying very little space. Then followed a neat battiste roll-up case, holding scissors, spring forceps, clinical thermometer, food thermometer, and two vaginal tubes. The case can be washed and sterilised. The dressing forceps are made so that the two pieces meeting at the top do not touch each other, but leave space for a towel or cloth to be inserted, so they can be thoroughly dried and no rust or dirt harboured in an otherwise unget-at-able quarter. A sterilisable enema syringe, a catheter in a waterproof case, soap and nail-brush box, powder and packets of gauze complete the contents of the steriliser. There is a patent wickless lamp to go with it, but unless it is especially wanted any moderate-sized spirit lamp (of which most nurses have one) would answer the purpose, and the steriliser can be had separately. The douche can mentioned is made of tin, holds two pints, and has folding feet; if desirable a small spirit lamp can be placed underneath, which would be a great convenience should the nurse have got everything ready and then be unavoidably detained. The cost of the steriliser with contents is £1 16s., the lamp 7s. 6d.

A point which impressed me was the way in which things were made to pack up and take as little space as possible, thus preventing overweight, and also how details had been considered to meet the many demands of aseptic surgery.

The midwives' bags can be had on approval from Messrs. Bailey. This affords a good opportunity for nurses to judge for themselves, and as they vary in price from 10s. to £2 19s. 6d. there must surely be one to meet most district nurses' needs and means. Any of the appliances can be bought separately.

### To Nurses.

We invite contributions from any of our readers, and shall be glad to pay for "Notes on News from the Nursing World," or for articles describing nursing experiences at home or abroad dealing with any nursing question from an original point of view, according to length. The minimum payment is 5s. Contributions on topical subjects are specially welcome. Notices of appointments, letters, entertainments, presentations, and deaths are not paid for, but we are always glad to receive them. All rejected manuscripts are returned in due course, and all payments for manuscripts used are made as early as possible after the beginning of each quarter.

### TRAVEL NOTES AND QUERIES.

By our Travel Correspondent.

Paris or Brussels for a Week (Sister Margaret).—Cheapest route to Paris, second class return from London *via* Newhaven and Dieppe, £2 2s. 3d. You must then add the fare to and from Buxton. The cheapest place I can recommend is the Hôtel de Londres et de Milan in the Rue St. Hyacinthe, in a very central position. Ask for rooms on the fourth floor. You must reckon expenses in the hotel at about 6s. per day each. Then there are tips which will come to about another 5s. or 6s. for the week each. You see, therefore, roughly what you will have left for sight-seeing and excursions. To Brussels, second class return from London, £1 10s. 11d. Hôtel de Bavière or Hôtel du Rhin would do for you; terms about the same as above.

Rules in Regard to Correspondence for this Section.—All questioners must use a pseudonym for publication, but the communication must also bear the writer's own name and address as well, which will be regarded as confidential. All such communications to be addressed "Travel Correspondent, 28 Southampton Street, Strand." No charge will be made for inserting and answering questions in the inquiry column, and all will be answered in rotation as space permits. If an answer by letter is required, a stamped and addressed envelope must be enclosed, together with 2s. 6d., which fee will be devoted to the objects of "The Hospital" Convalescent Fund. Ten days must be allowed before an answer can be published.

### Notes and Queries.

**REGULATIONS.**

**The Editor is always willing to answer in this column, without any fee, all reasonable questions, as soon as possible.**

**But the following rules must be carefully observed.**

**1. Every communication must be accompanied by the name and address of the writer.**

**2. The question must always bear upon nursing, directly or indirectly.**

**If an answer is required by letter a fee of half-a-crown must be enclosed with the note containing the inquiry.**

*Nurses' Association.*

(113) Will you kindly tell me the address of the "Royal Chartered Nurses' Association?—*E. N. R.*

You probably mean the Society of Chartered Nurses. The address is 14 Princes Street, Cavendish Square, W.

*Holiday Nurse..*

(114) Will you kindly tell me where to apply for a nurse (trained preferred) to leave in entire charge of two babies during the holidays, while the mother and elder children are away?—*E. I. P.*

Your best plan would be to advertise or reply to advertisements; or the matron of the Great Ormond Street Hospital for Children, W.C., might be able to supply you with a suitable nurse.

*Central Midwives Board.*

(115) Will you kindly explain if a Q.C.H. diploma is equivalent to C.M.B.; or is the latter necessary in addition to the former? if necessary shall I have to go through another examination to obtain C.M.B., or will the production of diploma be sufficient to get registered?—*T. S. W.*

Since March 31st last midwives have been required to pass the examination of the Central Midwives Board before they can be registered. For all particulars apply to the Secretary of the Central Midwives Board, 6 Suffolk Street, Pall Mall, S.W.

*Probationer.*

(116) I should be grateful to you if you could tell me how to become a probationer in a general hospital?—*G. H.*

See "How to become a Nurse." "The Nursing Profession: How and Where to Train."

*Training.*

(117) Will you kindly tell me if the training for probationers at —— Hospital would be considered good? They advertise so often for probationers that it makes one doubt whether it ranks well as a training school.—*E. C.*

We think you have confused the name of a hospital with that of a nurses' home, as there is no hospital of the name you mention in England. The nurses' home of that name is an excellent one.

*Rural Midwives Association.*

(118) Will you please let me know the address of the Rural Midwives Association?—*C. B.*

47 Victoria Street, London, S.W.

*Surgical Training.*

(119) Will you inform me where I can obtain three months' good surgical training, either free or for small fee. I have had 18 months' hospital training, and have done medical nursing privately for some time, but I want some good abdominal and general surgical work, and I do not feel able to pay £1 per week?—*G. D.*

Could you not arrange to return to the hospital where you had 18 months' training to get the surgical experience there?

*Names of Instruments.*

(120) Will you kindly tell me where I could send for or get a catalogue, showing, and giving names of instruments, as I have been away from hospital for some time and am anxious to know them again?—*Anxious.*

Write to the Manager of the Scientific Press, 29 Southampton Street, Strand, W.C.

**Handbooks for Nurses.**

| | Post Free. |
|---|---|
| "A Handbook for Nurses." (Dr. J. K. Watson.) ... | 5s. 4d. |
| "Nurses' Pronouncing Dictionary of Medical Terms." ... | 2s. 0d. |
| "Art of Massage." (Creighton Hale.) ... ... ... | 6s. 0d. |
| "Surgical Bandaging and Dressings." (Johnson Smith.) | 2s. 0d. |
| "Hints on Tropical Fevers." (Sister Pollard.) ... ... | 1s. 8d. |

Of all booksellers or of The Scientific Press, Limited, 28 & 29 Southampton Street, Strand, London, W.C.

### For Reading to the Sick.

"EYE HATH NOT SEEN."

"Eye hath not seen": yet man hath known and weighed
A hundred thousand marvels that have been:
What is it which (the Word of Truth hath said)
Eye hath not seen?

"Ear hath not heard": yet harpings of delight,
Trumpets of triumph, song and spoken word,
Man knows them all; what lovelier, lofter might
Hath ear not heard?

"Nor heart conceived": yet hath man now desired
Beyond all reach, beyond his hope believed,
Loved beyond death. What fire shall yet be fired
No heart conceived?

"Deep calls to deep": Man's depth would be despair
But for God's deeper depth. We sow to reap;
Have patience, wait, betake ourselves to prayer.
Deep answereth deep.

*C. Rossetti.*

The twin mysteries of eternal justice and eternal love are so deep and vast that we can stand before them only with an awful reverence; we can enter into them only with dread; but to see first the whole meaning of both in the sacred face of our Redeemer is to embrace both with a transcendent act of the will, which is faith and hope and love fulfilled in one simple Godward movement of being.

We know that growth and fruitfulness, discipline and correspondence with God bring pain in proportion to the ardour of the soul that willingly goes forward to suffer them; yet how glad and joyful are you even here in that pain which is the deep imprinting of the character of Christ, and of the idea of the Divine Will upon you.

How blest is that pain, preparing the soul and spirit for the possession of God! What wonderful growth and increase of grace, what unfolding of possibilities, what opening of capacities, when now the gifts of the Holy Spirit are at last free in their operation, when the powers of the Incarnate Life are fulfilled in the disposition of the whole man to the love of God.

We pass not to that which is unknown and dreadful, but to that for which even now we are being prepared by the unutterable working of the love of God within our own souls.

And when that future life becomes clearer to us, and clearer still as the years go on, surely something of its sweetness and power, something of the glory and mystery of it, will pass into our own lives to sweeten our relation with all the life of earth, changing the things which now are so full of disappointment into that which is certain and sure, because containing within itself all the promise, not only of the life that now is, but of that which is to come.

*Rev. G. Brett.*

Holy Saviour, calm our fears
When earth's brightness disappears.
Grant us in our later years
Light at evening time.

Holy Spirit, be Thou nigh
When in mortal pains we lie;
Grant us, 'ere we come to die,
Light at evening time.

*R. Hayes Robinson.*

